# Perceptions of Study Conditions and Depressive Symptoms During the COVID-19 Pandemic Among University Students in Germany: Results of the International COVID-19 Student Well-Being Study

**DOI:** 10.3389/fpubh.2021.674665

**Published:** 2021-06-10

**Authors:** Paula Mayara Matos Fialho, Franca Spatafora, Lisa Kühne, Heide Busse, Stefanie M. Helmer, Hajo Zeeb, Christiane Stock, Claus Wendt, Claudia R. Pischke

**Affiliations:** ^1^Institute of Medical Sociology, Centre for Health and Society, Medical Faculty, Heinrich Heine University Duesseldorf, Duesseldorf, Germany; ^2^Department Prevention and Evaluation, Leibniz-Institute for Prevention Research and Epidemiology – BIPS, Bremen, Germany; ^3^Institute of Health and Nursing Science, Charité – Universitätsmedizin Berlin, Berlin, Germany; ^4^Health Sciences Bremen, University of Bremen, Bremen, Germany; ^5^Sociology of Health and Health Care Systems, University Siegen, Siegen, Germany

**Keywords:** study conditions, university students, COVID-19, mental health, pandemic

## Abstract

**Background:** Results of previous studies examining the impact of the SARS-CoV-1 epidemic in 2003 on university students' mental well-being indicated severe mental health consequences. It is unclear how the current COVID-19 pandemic and the changes in study conditions due to federal regulations affected mental well-being in the German student population. We examined university students' perceptions of study conditions during the COVID-19 pandemic and investigated associations between study conditions and depressive symptoms.

**Methods:** A cross-sectional online survey was conducted in Germany in May 2020 at four universities (*N* = 5,021, 69% female, mean age: 24 years, *SD*: 5.1). Perceived study conditions, as well as sociodemographic information, were assessed with self-generated items and the CES-D 8 scale was used to determine depressive symptoms. Associations between perceived study conditions (academic stress and academic satisfaction), in general, and confidence to complete the semester, in particular, and depressive symptoms were analyzed using generalized linear regressions.

**Results:** Fifty-four percent of survey participants felt that the university workload had significantly increased since the COVID-19 pandemic; 48% were worried that they would not be able to successfully complete the academic year; 47% agreed that the change in teaching methods caused significant stress. Regarding depressive symptoms, the mean score of the CES-D 8 scale was 9.25. Further, a positive association between perceived study conditions and depressive symptoms was found (*p* < 0.001), indicating that better study conditions were associated with fewer depressive symptoms. Results of the generalized linear regression suggest that better student mental well-being was related to higher confidence in completing the semester.

**Conclusions:** This study provides first insights into perceived study conditions and associations with depressive symptoms among students during the COVID-19 pandemic in Germany. Findings underline the need for universities to provide intervention strategies targeting students' mental well-being during the course of the pandemic.

## Introduction

According to the Robert-Koch Institute (RKI), to date, in Germany, there are 2,434,446 confirmed cases of COVID-19 as of February 27^th^, 2021 (1). The necessary measures that were taken in order to minimize the spreading of COVID-19 affected the entire German society and many aspects of daily lives of German citizens. In mid-March 2020, universities were closed and lectures and courses were predominantly held online. New formats had to develop *ad hoc* by teaching staff because they had not been implemented in most universities prior to the outbreak (2).

It is known from previous research examining the consequences of epidemics on physical and psychological health that an epidemic outbreak seriously impacts on health (of those afflicted with the disease and those avoiding infection). Past epidemics, including SARS-CoV-1 that occurred in China in 2003, Ebola in 2014 in West Africa, and MERS in 2015, had a severe negative impact on individuals' mental well-being. Existing literature on the impact of the COVID-19 pandemic suggests that there is the potential of drastic psychological consequences in the general population, including University students (e.g., increases in the prevalence of depression and anxiety) (3). Taking into consideration that COVID-19 compared to e.g., the SARS-CoV-1-epidemic is a more infectious virus and more threatening to a larger part of the population and the fact that more people were in self-isolation or lockdowns, similar psychological consequences, as a result of the ongoing COVID-19 pandemic, are conceivable at the population level.

A large body of evidence indicates that college and University students are generally vulnerable to mental health disorders due to academic stressors, such as the pressure to succeed, uncertainty of plans after graduation, and financial worries experienced while studying (4–6). Previous research suggests that University students are more likely to develop general anxiety disorder or depression (7) or even suicidal thoughts and behaviors (8) when compared to individuals in the same age bracket but not enrolled in tertiary education. The fact that universities were closed and students had to get used to online education which was previously not part of most University culture may have increased students' concerns about not being able to successfully complete the academic year, accompanied by a negative impact on their mental well-being (9). Also, as everyday social interactions with fellow students in the University setting were no longer taking place, social isolation may have impacted on mental well-being. This situation may have also exacerbated existing psychological symptoms and the impact of stressors evident prior to the outbreak (10). While no investigations focusing on this particular population have yet been published, there have been reports on the psychological impact of the COVID-19 pandemic on young adults, indicating that younger individuals (aged <35 years) reported a higher prevalence of general anxiety disorder and depressive symptoms compared to older individuals (aged >35 years) (11). Further, previous research suggests that academic stress and poor academic satisfaction can be consequences of difficult study conditions (12). In our paper, the constructs of academic stress and academic satisfaction were combined as an indicator for study conditions (13).

Hence, to investigate possible consequences of the shift in study conditions during the first COVID-19 outbreak in March 2020 in Germany, we investigated perceptions of study conditions and depressive symptoms among University students in a cross-sectional study. This study aimed to elucidate possible associations of (changed) study conditions and the accompanying confidence to complete the semester with depressive symptoms among University students in various geographical regions of Germany using data from the International COVID-19 Student Well-being Study (C19 ISWS).

## Materials and Methods

### Study Design—The International COVID-19 Student Well-Being Study

This study is part of the C19 ISWS, examining the impact of the COVID-19 pandemic on student well-being at 110 higher education institutions in 27 European and North-American countries, as well as in South Africa. It is led and coordinated by a research team at the University of Antwerp in Belgium (UAntwerp, principal investigators: Sarah Van de Velde, Veerle Buffel, and Edwin Wouters). The study protocol outlining the aims and methods employed in the study can be found elsewhere (13).

The original version of the questionnaire was developed and distributed (in English) by the UAntwerp research team and two authors of the German team (SMH, HB) translated it into German. The UAntwerp research team inserted the translated questions into the Qualtrics software. Data collection using the online Qualtrics survey was carried out for 2 weeks (from May 12^th^ to 29^th^, 2020) at each participating German University. In the online survey, sociodemographic information, perceived study conditions, financial resources, health behavior, living situation before and during COVID-19 outbreak, COVID-19 diagnosis and symptoms, perceived worries, critical health literacy and knowledge about COVID-19, and mental well-being were assessed. The core questionnaire used can be found elsewhere (14).

### Recruitment at the German Study Sites and Participation

As part of the larger C19 ISWS, the online survey was conducted at four German universities in May 2020; participants included 5,021 students, approximately two-thirds of the sample came from the University of Bremen and the University of Siegen and the other third constituted students from the Charité—Universitätsmedizin Berlin and the Heinrich Heine University Duesseldorf (15). Students were predominantly invited to participate in the study via email and on the universities' homepages, as well as e-learning platforms. Invitations were also distributed via social media channels, such as Instagram or Facebook channels.

In May 2020, all the universities named above were closed in order to reduce the spreading of COVID-19. Most teaching was performed online. On-campus activities, such as campus sports, were prohibited and University canteens, cafés, and libraries were also closed at the time of data collection.

All participants provided their consent to participate prior to completing the survey. An informed consent page containing the research objectives, information on data security, subjects' privacy, confidentiality, non-material incentive, and the contact details of the researcher, preceded the survey. Participation in the survey was voluntary, and individuals could withdraw at any time during the survey by closing the web browser.

Ethical approval for conducting the study was obtained at the Ethics Committees of the four participating universities.

### Measures

#### Perceived Study Conditions

In this article, two self-constructed scales which were based on factor analysis were used to assess perceived study conditions; one measuring academic stress and the other academic satisfaction (16). Students were asked to indicate to what extent they agreed with the following eight statements:

(1) “My university/college workload has significantly increased since the COVID-19 outbreak;” (2) “I know less about what is expected of me in the different course modules/units since the COVID-19 outbreak;” (3) “I am concerned that I will not be able to successfully complete the academic year due to the COVID-19 outbreak;” (4) “The university/college provides poorer quality of education during the COVID-19 outbreak as before;” (5) “The change in teaching methods resulting from the COVID-19 outbreak has caused me significant stress;” (6) “The university/college has sufficiently informed me about the changes that were implemented due to the COVID-19 outbreak;” (7) “I am satisfied with the way my university/college has implemented protective measures concerning the COVID-19 outbreak;” (8) “I feel I can talk to a member of the university/college staff (e.g., professor, student counselor) about my concerns due to the COVID-19 outbreak.” Response categories forming a 5-point Likert scale ranged from strongly agree to strongly disagree. The perceived study conditions were assessed as a sum of the ratings, with reverse coding of the positive items, so that a higher score of study conditions indicates higher academic stress and dissatisfaction and a lower score lower stress and higher satisfaction. The score was used as a continuous variable.

To assess the frequency of contact with teaching staff, students were asked the following question: “In comparison to the period before the COVID-19 outbreak, did you seek more or less contact with the teaching staff at your university/college:” (a) to discuss worries about studies, (b) to discuss psychosocial problems. Response options ranged from much less to much more on a 5-point Likert scale. We combined the categories “much less” with “less” and “more” with “much more.”

To assess the contact with student-counseling and social services, students were asked “Since the COVID-19 outbreak, did you seek contact with student-counseling services or social services at your university/college?” (response options: yes/no). The following reasons could be named for contacting counseling services (multiple choice): worries about studies; financial worries/difficulties; psychosocial problems; other worries; prefer not to say.

#### Subjective Depressive Symptoms

Subjective depressive symptoms were assessed using the Center for Epidemiological Studies Depression Scale (CES-D 8 scale) (17, 18). The eight items of the CES-D 8 tracked whether students felt depressed and that everything was an effort, had restless sleep, could not get going, felt lonely, or sad or enjoyed life and felt happy (the last two items are reverse-coded). Students were asked to indicate how much of the time during the past week the symptoms listed above applied to them on a 4-point Likert scale [(0) none or almost none of the time; (1) some of the time; (2) most of the time; (3) all or almost all of the time]. Responses to the individual items were summed up to create the overall CES-D 8 score. The score can range from 0 to 24, a higher score suggesting higher levels of depressive symptoms.

#### Covariates

The following information on the socio-demographic characteristics of students was provided: age, gender (female/male/diverse), relationship status (“single”/“in a relationship”/“it is complicated”), resident status in Germany (permanent residency/temporary residency), availability of a person to discuss intimate matters with (yes/no) were collected.

In regard to their study program, students were asked which study program they were enrolled in [Bachelor program/Master Program/Doctoral Program/State Examination (Medicine, Law)/other].

The following information was provided regarding the living situation: Students were asked where they mainly lived during the COVID-19 outbreak (excluding weekends and holidays). The response options were: with parents/student hall/accommodation with others/accommodation alone and other.

Concerning their financial situation, students were asked to indicate whether they had sufficient financial resources to cover their monthly expenses or not during the COVID-19 outbreak.

### Data Analysis

Descriptive statistics were calculated to summarize the sample, regarding the sociodemographic data and study related information. Absolute (*n*) and relative (%) frequencies were determined for perceived study conditions (academic stress and academic satisfaction) and subjective depressive symptoms during the COVID-19 outbreak using univariate analysis. The frequencies of contact with teaching staff and with student counseling and social services and reasons for getting in touch with these services were descriptively analyzed. Generalized linear regression analyses were conducted to identify the associations between the dependent variable depressive symptoms and the independent variables. Perceived study conditions (academic stress and academic satisfaction), age, gender, program enrolled in, relationship status, living situation, availability of a person to discuss intimate matters with, financial resources, resident status, and study site were included as independent variables in the model. In order to assess confidence in being able to complete the semester, an additional analysis was performed using one item of the academic stress scale (able to successfully complete the academic year due to the COVID-19 outbreak). The association of the confidence to complete the semester and depressive symptoms was examined with a generalized linear regression using the same covariates listed above.

Data analysis was performed using IBM® SPSS® version 26 and SAS Version 9.4 (SAS Institute, Cary, NC, USA).

## Results

A total of 5,021 students completed the online survey: 69.4% were female and 29.4% male and the mean age was 24 years (*SD* = 5.1; Min = 17; Max = 50). A total of 53.8% of students were enrolled in a Bachelor program. One-third of the students were living with their parents at the time of data collection. The study sample is described in further detail in Table 1.

**Table 1:** Sociodemographic and study related characteristics of participants.

	***n***	**%**
**AGE**		
17–19	369	7.3
20–23	2,340	46.6
24–27	1,396	27.8
27–30	454	90
>30	462	9.2
**GENDER**		
Male	1,478	29.4
Female	3,485	69.4
Diverse	58	1.2
**STUDY PROGRAM**		
Bachelor program	2,700	53.8
Master program	1,138	22.7
Doctoral program	234	4.7
State examination (Medicine, Law)	910	18.1
Other	39	0.8
**RELATIONSHIP STATUS**		
In a steady relationship	2,677	53.3
Single	2,149	42.8
It is complicated	195	3.9
**LIVING SITUATION**		
With parents	1,973	33.3
Student hall	284	4.8
Accommodation with others	2,067	24.9
Accommodation alone	611	10.3
Other	115	1.9
**PERSON TO DISCUSS INTIMATE MATTERS WITH**		
Yes	4,478	90.4
No	474	9.4
**FINANCIAL RESOURCES**		
Sufficient to cover monthly costs	4,277	85.2
Not sufficient to cover monthly costs	744	14.8
**STATUS IN GERMANY**		
Permanent residency	4,725	94.1
Temporary residency	296	5.9

No significant differences in study conditions (and perceived depressive symptoms) by gender were found in this study. In the following sections, results are therefore reported combining the data for men, women, and diverse students.

Regarding the subjective depressive symptoms during the COVID-19 pandemic, approximately 31% of participants felt frustrated with things, in general. Thirty-one percent of students felt isolated from others. One in four students stated that they felt depressed all or almost all/most of the time during the COVID-19 outbreak. The mean score on the CES-D 8 scale was 9.25 (Table 2).

**Table 2:** Description of subjective depressive symptoms during the outbreak of COVID-19 (*n* = 4,933).

**Items of CES-D 8 scale**	**All or almost all/most of the time**		**Some of the time**		**Almost none or none of the time**	
	***n***	**%**	***n***	**%**	***n***	**%**
Felt depressed	1,240	24.7	2,541	50.6	1,152	22.9
Felt that everything they did was an effort	1,489	29.7	2,046	40.7	1,398	27.8
Sleep was restless	1,447	28.6	2,064	41.1	1,422	28.3
Happy	2,540	50.6	2,060	41.0	333	6.6
Felt lonely	994	19.8	1,916	38.2	2,023	40.3
Enjoyed life	2,105	42.0	2,177	43.4	651	13
Felt sad	920	18.3	2,683	53.4	1,330	26.5
Could not get going	1,440	28.7	2,033	40.5	1,460	29.1
		**Mean**	**95% CI**	**SD**		
**CES-D 8 Scale**		9.25	9.11–9.38	0.67		

*CI, confidence interval; SD, standard deviation*.

In Table 3, results regarding students' perceived study conditions are displayed. Around half of students felt that the university/college workload had significantly increased since the COVID-19 outbreak. Fifty-four percent stated they knew much less what was expected of them in the different courses/units; 47.9% were worried that they would not be able to successfully complete the academic year; 47.2% agreed that the change in teaching methods caused them significant stress.

**Table 3:** Perceived study conditions among University students (*n* = 4,903).

	**Strongly agree/Agree**		**Neither agree nor disagree**		**Disagree/Strongly disagree**	
	***n***	**%**	***n***	**%**	***n***	**%**
University/college workload significantly increased	2,741	54.6	1,073	21.4	1,089	21.8
Knowing less about what is expected of me in the different course modules/units	2,713	54.1	958	19.1	1,232	24.5
Concerned about not being able to successfully complete the academic year	2,409	47.9	701	14.0	1,793	35.7
University/college provides poorer quality of education	1,756	35.0	1,455	29	1,692	33.7
Change in teaching methods caused significant stress	2,371	47.2	928	18.5	1,604	31.9
University/college provided sufficient information about the implemented changes	3,515	70.0	656	13.1	732	15.4
Satisfied with the implemented protective measures taken by University	3,341	66.5	932	18.6	630	12.6
Feel that one can talk to a member of the university/college staff (e.g., professor, student counselor)	1,591	31.7	1,962	39.1	1,350	26.9

Table 4 shows students' contact to teaching staff and student counseling or social services at their University. Respondents who generally sought contact with teaching staff to discuss worries about studies or psychosocial problems reported that they had less done so in comparison to the time before the outbreak. Merely 5% of study participants sought contact with student counseling or social services at University. They predominantly used the services to discuss worries about their studies.

**Table 4:** Frequency of contact with teaching staff and contact with student-counseling services or social services and reasons for contact (multiple choice).

	**Much less/Less**		**Similar**		**More/Much more**	
	***n***	**%**	***n***	**%**	***n***	**%**
Contact with teaching staff to discuss worries about studies (*n* = 2,196)	1,020	46.4	699	31.8	477	21.7
Contact with teaching staff to discuss psychosocial problems (*n* = 1,174)	565	48.1	514	43.8	95	8.1
	**Yes**			**No**		
	***n***		**%**	***n***		**%**
Contact with student-counseling services or social services (*n* = 4,928)	251		5.1	4,677		94.9
Reasons for contact with student-counseling services or social services	***n***		**%**			
Worries about studies	127		50.6			
Financial worries/difficulties	46		18.3			
Psychosocial problems	6		2.4			
Other worries	71		28.3			
Prefer not to say	31		12.4			

The multiple regression model, adjusted for the selected covariates, revealed that higher academic stress was associated with a higher score on the CES-D 8 scale (*p* < 0.0001). Living with parents or living in an accommodation with other people was associated with lower levels of symptoms of depression, as well as having sufficient financial resources. Moreover, University students in a steady relationship (compared to being single) and those indicating the presence of a person to discuss personal matters with (compared to not having such a person in their lives) also more likely to report lower symptoms of depression. No differences in depressive symptoms were observed by the program students were enrolled in or by age (Table 5).

**Table 5:** Effect estimates of the generalized linear model of study conditions on depressive symptoms.

	**Regression on depressive symptoms score**	
**Variable**	**Regression coefficient^**a**^ (*SD*)**	***p*-Value**
*Intercept*	7.81 (1.09)	<0.0001
Study conditions	0.25 (0.01)	<0.0001
Age	0.00 (0.01)	0.8208
Gender		
Female	0.00 (0)	*Reference*
Male	−1.03 (0.14)	<0.0001
Diverse	2.31 (0.58)	<0.0001
Study program		
Bachelor programme	0.00 (0)	*Reference*
Master programme	0.21 (0.72)	0.7680
Doctoral programme	−0.12 (0.72)	0.8692
State examination (Medicine, Law)	−0.29 (0.76)	0.6998
Other	−0.40 (0.72)	0.5748
Relationship status		
In a steady relationship	−0.70 (0.14)	<0.0001
Single	0.00 (0)	*Reference*
It's complicated	0.93 (0.32)	0.0037
Living situation		
With parents	−0.90 (0.21)	<0.0001
Student hall	−0.41 (0.32)	0.1979
Accommodation with others	−0.76 (0.20)	0.0002
Accommodation alone	0.00 (0)	*Reference*
Other	−0.81 (0.44)	0.0659
Person to discuss intimate matters with		
Yes	−3.18 (0.21)	<0.0001
No	0.00 (0)	*Reference*
Financial resources		
Sufficient to cover monthly costs	−2.02 (0.18)	<0.0001
Not sufficient to cover monthly costs	0.00 (0)	*Reference*
Status in Germany		
Permanent residency	0.00 (0)	*Reference*
Temporary residency	0.35 (0.28)	0.2060

a *Adjusted for age, gender, study program, relationship status, living situation, person to discuss intimate matters with, financial resources, residency status, study site*.

The regression model analysing confidence in being able to complete the semester and depressive symptoms yielded a higher regression coefficient for the CES-D 8 score, the more concerned students were about being able to complete the semester. Conversely, the more confident students were in being able to complete the semester, the better their depressive symptoms (Table 6).

**Table 6:** Effect estimates of the generalized linear regression model of confidence in completing the semester on depressive symptoms.

	**Regression on depressive symptoms score**	
**Variable**	**Regression coefficient^**a**^ (*SD*)**	***p*-Value**
*Intercept*	12.34 (1.03)	<0.0001
Able to successfully complete the academic year due to the COVID-19 outbreak		
Strongly agree	4.46 (0.21)	<0.0001
Agree	2.68 (0.20)	<0.0001
Neither agree nor disagree	2.11 (0.22)	<0.0001
Disagree	1.12 (0.20)	<0.0001
Strongly disagree	0.00 (0)	*Reference*

a *Adjusted for age, gender, study program, relationship status, living situation, person to discuss intimate matters with, financial resources, residency status, study site*.

## Discussion

This paper investigated perceptions of study conditions during the first COVID-19 outbreak and the associations with students' depressive symptoms. We found that approximately half of the students felt burdened by an increased workload and felt worried about being able to successfully complete the academic year. Both of these factors are indicators for an increase in students' academic stress. Further, our results suggest that higher academic stress and dissatisfaction were associated with higher levels of depressive symptoms.

### Depressive Symptoms

The high level of depressive symptoms among University students, which we found in our study (CES-D 8 means 9.25), was similar to the findings among Belgian higher education students (CES-D 8 means 10.8) (16). Also, Li et al. reported an increase in negative affect and symptoms of anxiety and depression since the COVID-19 outbreak among University students due to the restrictions in the University context (19). Another study that investigated the psychological impact of COVID-19 in Chinese University students reported an increase of symptoms of anxiety among students (20). Further, a study conducted at a University in the United States of America found alarming results concerning students' mental health enrolled in higher education under COVID-19 conditions (21). They found an increase of anxiety and stress due to the COVID-19 outbreak for the majority of the students who completed the survey (71%). This study identified various stressors similar to the ones assessed in our study contributing to increased levels of stress (21).

### Study Conditions and Mental Well-Being

COVID-19 and its various consequences have an impact and will continue impacting on University students' mental health and well-being (22) and the study conditions are just one of many factors that have changed for students since the COVID-19 outbreak. Previous studies showed that worrying about academic performance is a stressor among University students (21). This leads to an increase of academic stress and it is known from previous literature that academic stress is associated with depression and anxiety (4). Accordingly, high levels of academic stress (academic expectations, faculty work and examinations, students' academic self-perceptions) were reported by Italian University students during the COVID-19 outbreak in a study (9) that also found a negative correlation between academic stress and mental well-being during the pandemic.

### Contact With Student's Services and Students' Confidence

Our results indicate that students rather sought less contact to teaching staff than before the COVID-19 outbreak and very few had contact to student counseling services. Many students felt that, since the outbreak, their workload for University had increased, as well as their fear of not being able to successfully finish the semester. Furthermore, the results of our study indicate that the higher students' confidence to complete the semester, the better their depressive symptoms. This is plausible as students experienced delays in their academic progress and possibly an impact on future employment due to the COVID-related restrictions (23).

### Social Distance and Social Support

Previous research demonstrated the negative impact of quarantine on students' mental health (24). Quarantine was identified as a stressor among Pakistani medical students reporting that they felt emotionally detached from their families and friends (24). Also, evidence suggests that social distancing affected the mental well-being of students negatively (25). This is in line with our finding that living together with other people is associated with better mental well-being. In a stressful situation, such as the COVID-19 pandemic, social support plays an important role for maintaining mental well-being and reliable people can help maintain mental well-being (26, 27). Social support can come in various forms (28) and having someone to discuss personal matters with or living with parents could be protective factors against the development of psychological problems. Even in times of social distancing, promoting alternatives for this in-person support and exchange may help reduce stress and improve students' mental well-being.

The results of our study call for several actions, which should be undertaken by universities to minimize harm on the part of students. Universities should provide easily accessible student counseling and social services, as well as mental health care services. Moreover, continuous evaluation of students' perceptions of study conditions are needed to adjust information flow and academic practices to student's needs. Because this global pandemic has led to a global mental health crisis (29), universities should be aware of the increased risk of mental health problems among their student body, especially taking into consideration that the global student population was already considered a group vulnerable to mental illness prior to the outbreak (30). Building institutional and societal awareness of students‘ needs for mental health care is important in order to support them (22). Hence, universities should provide timely and appropriate mental health care to students in the future. The findings of the recent studies show the urgent need to develop interventions and preventive strategies to address the mental health of college students in the current situation (21). Therefore, universities should develop strategies to identify and support students at higher risk for negative psychological consequences during the COVID-19 pandemic (31). Digital mental health care can be an alternative to support students and improve their mental well-being, especially, in situations where in-person counseling is provided due to COVID-19-related restrictions (32, 33). Training for faculty preparing them for shifts to online teaching is necessary in this process as well (34). As much of the students' perceived stress came from the insecurity of the educational situation, universities can reduce stress by ensuring smoother transitions in the future.

Some limitations need to be addressed. In the International COVID-19 Student Well-being Study, depressive symptoms were assessed with a shortened version of the validated CES-D scale, first validated in a sample of older adults (35). The perceived study conditions refer to the time since the outbreak only. Therefore, we could not investigate how students perceived the study conditions before the outbreak in comparison to the time during the pandemic. A study conducted in October 2017 to March 2018 in Germany, in a not representative sample, found that perceived academic stress explained a great amount of distress symptoms among University students (36). In addition, the study compares the pandemic study conditions in some of the items with time before the outbreak, but, unfortunately, depressive symptoms were only recorded for the time during the outbreak. Another study among University students in Germany compared the impact of lockdown stress and loneliness during the COVID-19 pandemic on mental health and found a higher level of depressive symptoms during than before the pandemic (38.5 vs. 27.7%) (37). Our results suggest that approximately one-half of the assessed student population felt that study conditions had worsened during the COVID-19 outbreak and that this might have had a negative impact on their mental well-being. However, the results are limited due to the cross-sectional design of the study and the causality of the findings remain unclear. Longitudinal studies are necessary to examine the impact of changed study conditions on mental well-being in the long-run. Furthermore, the response rate was limited to approximately 10% and selection bias cannot be ruled out.

To conclude, this study provides first insights into associations between perceived study conditions and depressive symptoms among University students during the COVID-19 pandemic in Germany. Additional research is necessary to examine mental well-being of students under pandemic conditions in the long-term and to evaluate whether strategies developed by universities to help students cope with the situation are successful or not.

## Data Availability Statement

Due to the nature of this research, participants of this study did not agree for their data to be shared publicly, so supporting data are not publicly available. Data are available on request from the corresponding author for collaborating researchers within the C19 ISWS consortium, as consent for this was provided from all participants.

## Ethics Statement

The studies involving human participants were reviewed and approved by the Ethics Committee of all four participating universities (Charité—Universitätsmedizin Berlin, University of Bremen (protocol code 2020-04-EILV, dated 4 May 2020), Heinrich-Heine University Duesseldorf (protocol code 2020-958, dated 5 May 2020) and the University of Siegen (protocol code ER 08/2020, dated 7 May 2020). The patients/participants provided their written informed consent to participate in this study.

## Author Contributions

PMMF, FS, and CRP developed the study questions for this investigation. PMMF, FS, and LK conducted the statistical analyses and wrote the first draft of the article. All authors were involved in the conception and implementation of the German survey and critically revised the content of the article. All authors contributed to the article and approved the submitted version.

## Conflict of Interest

The authors declare that the research was conducted in the absence of any commercial or financial relationships that could be construed as a potential conflict of interest.
